# Supplementary material for: Scissor Bite in Growing Patients: Case Report Treated with Clear Aligners
Source: Children (Basel). 2023 Mar 27;10(4):624. doi: 10.3390/children10040624 (PMC10136876; doi:10.3390/children10040624)
Supplement: Supplementary file 1 [file children-10-00624-s001.zip › children-2259405-supplementary.pdf]

children MDPI

## Supplementary materials

**Table S1.** Different conventional approach to treat SB in growing patients, according to literature review.

| Author and year of publication | Title | Sample | Etiology | Treatment | Conclusion |
| --- | --- | --- | --- | --- | --- |
| King J. et al, 2004 [20] | "Unilateral Brodie bite treated with distraction osteogenesis" | ♀11Y and 10M<br>SB- left side (24,25,26/34,35, 36)<br>**Other associated problems:** Convex profile without facial asymmetry | SB- skeletal (mandible)<br>Right molar and canine Class I<br>Skeletal Class II, hyperdivergent | **<u>Maxilla:</u>** fixed appliance + full coverage maxillary appliance<br>**<u>Mandible:</u>** fixed appliance + medial symphysis distraction osteogenesis with hybrid distractor<br>**<u>Total treatment time:</u>** 2Y and 2M<br>**<u>Retainer:</u>** Hawley's removable<br>**<u>Control:</u>** 1Y and 6 M, minimal skeletal relapse post-distraction | Distraction osteogenesis has shown to be minimally invasive, comfortable, predictable, and affordable. |
| Yun S. et al, 2007 [19] | "Scissors-bite correction on second molar with a dragon helix appliance" | ♀ 16Y<br>SB- left side (27/37)<br>**Other associated problems:** Convex profile | SB- dental<br>Bilateral Class II<br>Skeletal Class I | **<u>Maxilla:</u>** 1 mini-screw (MS) between 25 and 26 connected through stainless steel wire to 26B + dragon helix spring (1 arm 27O, 1 arm 26B)<br>**<u>Mandible:</u>** 1MS between 35 and 36 connected to 36B + bracket per lingual of 37 with elastic bands<br>**<u>SB Correction:</u>** 10M | A spring dragon helix combined with indirect skeletal anchorage was used to successfully treat a SB. |
| Nojima K.et al, 2010 [2] | "A case report of Bilateral Brodie Bite in Early Mixed Dentition Using Bonded Constriction Quad-helix Appliance" | ♂ 9Y and 2M<br>SB- bilateral posterior<br>**Other associated problems:** Convex profile and mandibular retrognathism | SB-skeletal (maxilla and mandible)<br>Bilateral molar Class II<br>Skeletal Class II | **<u>Maxilla:</u>** quad-helix appliance (constriction) with resin on O-face of 16,55,54,64,65,26<br>**<u>Mandible:</u>** bi-helix appliance on 36 and 46 (expansion)<br>**<u>Total treatment time:</u>** 2Y and 6M<br>**<u>SB Correction:</u>** 5M | Treatment of bilateral SB in the mixed dentition with a quad helix appliance provided an effective reduction in maxillary width, providing good results in a short period of time and without patient´s cooperation |

| Author and year of publication | Title | Sample | Etiology | Treatment | Conclusion |
|---|---|---|---|---|---|
| Pinho T.,2011 [1] | "Early treatment of scissor bite" | **Case 1**: ♂ 6Y SB- right side (54,55,16/84,85,46) Hypodivergent | **Case 1:** SB- Dental, compressed mandible Right molar Class III Left molar Class I | **Case 1:** **<u>Mandible:</u>** cemented expansion appliance with bite plate **<u>Retainer</u>**: same expander for 1 month **<u>SB correction:</u>** 4M **<u>Control:</u>** 6M, no relapse | Mandibular dental expansion appliance with bite plates and intra and inter arch elastics as an effective combination. |
| | **Case 2:** ♂ 8Y SB-right side (54,55,16/84,85,46) Hypodivergent | **Case 2:** SB-dental Maxilla greatly expanded and Compressed mandible Bilateral molar Class I | **Case 2:** **<u>Maxilla:</u>** crossed elastics between 16 and 55 and mandibular expander + intra-arch elastics between 16 and 26 and 55 and 65 **<u>Mandible:</u>** cemented expansion appliance with bite plate + resin on 46 O and B **<u>SB Correction</u>**: 8M **<u>Control:</u>** 1Y, no relapses | **Case 2:** ♂ 8Y SB-right side (54,55,16/84,85,46) Hypodivergent | Anterior bite plate and elastics as an effective combination. |
| | **Case 3:** ♂ 12Y SB- left side (26/36) Hypodivergent | **Case 3:** SB-dental Right molar Class I | **Case 3:** **<u>Maxilla:</u>** Cross elastics used between 26 and 36. **<u>Mandible:</u>** lingual arch with bite plate except 36. **<u>SB correction:</u>** 3M **<u>Control:</u>** 3M, no relapse | **Case 3:** ♂ 12Y SB- left side (26/36) Hypodivergent | |
| | **Case 4:** ♀ 11Y SB- right side (14,15/44,45) Hypodivergent | **Case 4:** SB-dental Bilateral molar Class II | **Case 4:** **<u>Maxilla:</u>** bite-tubes in palatal of 11 and 21 + crossed elastic between 14 and 44 **<u>Mandible:</u>** resin per occlusal and buccal of 44 **<u>Total treatment time:</u>** 8M **<u>Retainer:</u>** bite tubes until eruption of the second molars | **Case 4:** ♀ 11Y SB- right side (14,15/44,45) Hypodivergent | |
| Hua X. et al, 2012 [14] | "Correction of a dental arch-width and asymmetric discrepancy with a slow maxillary contraction appliance" | ♂ 12Y and 3M SB- right side (14,15,16/44,45,46) **Other associated problems:** Convex profile and facial asymmetry | SB- skeletal (maxilla) Bilateral Molar Class III Bilateral Canine Class I Skeletal Class I | **<u>Maxilla:</u>** slow maxillary contraction removable appliance **<u>Total treatment time:</u>** 17M **<u>SB Correction:</u>** 7M **<u>Retainer:</u>** 1- the same appliance without the screw (5 months). 2- Hawley removable for maxilla and mandible **<u>Control:</u>** 37M, no relapses | Slow maxillary contraction is an inexpensive and effective way to treat transverse maxillary asymmetry. |

| Author and year of publication | Title | Sample | Etiology | Treatment | Conclusion |
|---|---|---|---|---|---|
| Chandorikar H. et al, 2017 [15] | "Early correction of unilateral scissor bite using transforce appliance and modified twin block appliance" | ♂ 9Y<br>SB- right side (54,55,16/84,85, 46)<br>**Other associated problems:** Straight profile, functional deviation to the left side | SB-dental<br>Left molar Class I<br>Bilateral canine Class III<br>Skeletal Class I | **Maxilla:** anterior bite plate<br>**Mandible:** 28 mm lingual transforce appliance, size 2<br>**Total treatment time:** 3Y and 6M<br>**SB Correction:** 5M<br>**Retainer:** lower lingual retainer + wraparound to the maxilla<br>**Control:** 2Y, no relapse | This early treatment approach can give predictable results with less patient dependence and discomfort. |
| Lee K. et al, 2020 [16] | "Scissor Bite Correction with TADs" | ♀ 16Y<br>SB-right side (16.17/46.47)<br>**Other associated problems:** Convex profile without facial asymmetry. | SB- dental<br>Left and right Class II<br>Skeletal Class I | **Maxilla:** fixed appliance with 2 MS in the midpalatal area and TPA + occlusal ramp on 26 and 15<br>**Mandible:** fixed appliance with 2 MS (1 in B between 45 and 46, 1 by DB of 47)<br>**Total treatment time:** 2Y and 5M<br>**SB Correction:** 2Y<br>**Retainer:** fixed lingual retainers in maxilla and mandible<br>**Control:** 6M, no relapse | MS can be used to successfully correct the SB. |
| Agrawal A., 2020 [6] | "Brodie Bite: A Clinical Challenge" | ♂ 13Y<br>SB- bilateral posterior<br>**Other associated problems:** Convex profile | SB-skeletal (mandible)<br>Bilateral Class II<br>Skeletal Class II | **Maxilla:** preadjusted edgewise appliance (initial phase) + Herbst appliance<br>**Mandible:** edgewise fixed appliance + Herbst appliance + lingual arch to the second molars<br>**Total treatment time:** 22M<br>**Retainer:** permanent lingual adhesives on the mandible and invisible on the upper arch | The Herbst appliance successfully corrected the SB with the advantages of reduced time and not needing the patient's cooperation. |

*__Legend__: SB- Scissor bite; Y- years; M- months; MS- mini-screw; B- buccal; O- occlusal; TPA- transpalatal arch; DB- distobuccal;*
